# White matter structures associated with loneliness in young adults

**DOI:** 10.1038/srep17001

**Published:** 2015-11-20

**Authors:** Seishu Nakagawa, Hikaru Takeuchi, Yasuyuki Taki, Rui Nouchi, Atsushi Sekiguchi, Yuka Kotozaki, Carlos Makoto Miyauchi, Kunio Iizuka, Ryoichi Yokoyama, Takamitsu Shinada, Yuki Yamamoto, Sugiko Hanawa, Tsuyoshi Araki, Hiroshi Hashizume, Keiko Kunitoki, Yuko Sassa, Ryuta Kawashima

**Affiliations:** 1 Department of Psychiatry, Tohoku Pharmaceutical University, Sendai, Japan; 2 Department of Functional Brain Imaging, Institute of Development, Ageing and Cancer, Tohoku University, Sendai, Japan; 3 Division of Developmental Cognitive Neuroscience, Institute of Development, Ageing and Cancer, Tohoku University, Sendai, Japan; 4 Division of Medical Neuroimaging Analysis, Department of Community Medical Supports, Tohoku Medical Megabank Organization, Tohoku University, Sendai, Japan; 5 Department of Nuclear Medicine and Radiology, Institute of Development, Ageing and Cancer, Tohoku University, Sendai, Japan; 6 Human and Social Response Research Division, International Research Institute of Disaster Science, Tohoku University, Sendai, Japan; 7 Smart Ageing International Research Center, Institute of Development, Ageing and Cancer, Tohoku University, Sendai, Japan; 8 Department of Adult Mental Health, National Institute of Mental Health, National Center of Neurology and Psychiatry, Kodaira, Tokyo, Japan; 9 Graduate School of Arts and Sciences, Department of General Systems Studies, The University of Tokyo; 10 Department of Psychiatry, Tohoku University Graduate School of Medicine, Sendai, Japan; 11 Japan Society for the Promotion of Science, Tokyo, Japan; 12 Faculty of Medicine, Tohoku University, Sendai, Japan

## Abstract

Lonely individuals may exhibit dysfunction, particularly with respect to social empathy and self-efficacy. White matter (WM) structures related to loneliness have not yet been identified. We investigated the association between regional WM density (rWMD) using the UCLA Loneliness Scale in 776 healthy young students aged 18–27 years old. Loneliness scores were negatively correlated with rWMD in eight clusters: the bilateral inferior parietal lobule (IPL), right anterior insula (AI), posterior temporoparietal junction (pTPJ), left posterior superior temporal sulcus (pSTS), dorsomedial prefrontal cortex (dmPFC), and rostrolateral prefrontal cortex (RLPFC). The bilateral IPL, right AI, left pSTS, pTPJ, and RLPFC were strongly associated with Empathy Quotient (EQ), whereas the bilateral IPL, right AI, left pTPJ, and dmPFC were associated with General Self-Efficacy Scale (GSES) score. The neural correlates of loneliness comprise widespread reduction in WMD in areas related to self- and social cognition as well as areas associated with empathy and self-efficacy.

Loneliness is an unpleasant state experienced when a discrepancy exists between the interpersonal relationships one wishes to have and those that one perceives oneself as currently having^1^. Loneliness is widespread^2^, particularly among young people^3^.

Empathy and self-efficacy appear to be critical factors related to loneliness. Loneliness is inversely correlated with empathy in young adults, while the social-skills factor in the Empathy Quotient (EQ) is a strong predictor of loneliness^4^. Self-efficacy, a person’s beliefs about his/her ability to complete tasks that impact the course of his/her life^5^, is negatively correlated with loneliness^6^.

On the physiological level, loneliness is associated with increased tonic sympathetic tonus, immunological impairment, reduced sleep quality, and neuroendocrine changes such as hypothalamic–pituitary–adrenocortical activation and the expression of genes regulating glucocorticoid responses in humans^3^,^7^. Clinically, the feeling of loneliness is associated with stress-related physical illness (e.g., cardiovascular disorders, obesity, and headaches)^2^,^3^. Thus, understanding the physiological underpinnings and neural correlates of loneliness can contribute to improved health and quality of life.

A recent neuroimaging study using voxel-based morphometry (VBM) found that lonely individuals had less grey matter (GM) in the left posterior superior temporal sulcus (pSTS) compared to non-lonely individuals. The study also demonstrated that social-perception skills mediated the association between loneliness and GM in left pSTS^8^. Importantly, changes in white matter (WM), including regional WM density (rWMD) and values for fractional anisotropy (FA), may be associated with changes in myelination, axonal membrane thickness, and the diameter and parallel organisation of axons^9^. Moreover, axonal calibre and myelin sheath thickness determine neuronal conduction velocity, which can alter cognitive function^10^. Thus, changes in both GM and WM structures in regions associated with loneliness may provide unique insights, reflecting changes in transmission across neural networks. However, the WM structures associated with loneliness have not yet been identified.

Because empathy is associated with loneliness, understanding the neural networks that underlie empathy could be a key factor in identifying the networks associated with loneliness. In a previous study^10^, we found that WM structures involving the default mode network (e.g., medial prefrontal cortex [mPFC], various areas of the temporal lobes, STS, and the temporoparietal junction [TPJ]) and the mirror neuron system (such as the inferior parietal lobule [IPL] and insula) were involved in the mediation of empathy. This finding was consistent with previous studies^11^. Thus, we hypothesised that the degree of loneliness would be negatively correlated with rWMD in those areas.

Furthermore, we predicted that the neural correlates of loneliness would be associated with the correlates of self-efficacy, as self-efficacy is a significant predictor of loneliness^6^. Self-efficacy is the belief in one’s ability to cope with a broad range of stressful or challenging demands^12^. Interestingly, self-efficacy and self-esteem are highly correlated with each other^13^. Importantly, lonely people had lower self-esteem than did non-lonely subjects^14^. Self-esteem and loneliness exerted a particularly strong influence on one another, with the dominant path from self-esteem to loneliness being mediated by perceived social acceptance^14^. Hence, self-esteem might also be an essential factor related to loneliness. We should also emphasize the role of self-reflection, as positive self-efficacy and self-esteem are attained through self-reflection^15^. Based on neuroimaging studies, lower self-esteem has been reported to have a linear relationship with activity in the ventral ACC and mPFC in response to positive versus negative social feedback^16^. Self-reflection is associated with activation of the dorsomedial and dorsolateral PFC (dmPFC, dlPFC), insula, anterior/posterior cingulate cortex (ACC, PCC), and left IPL^17^. Moreover, the dmPFC, TPJ, and pSTS were found to be involved in self-reflection. Additionally, the anterior insula (AI) plays a critical role in self-efficacy as it relates to the experience of negative emotions such as pain and disgust^11^ as well as to distress about rejection^18^ that one either personally experiences or observes in other individuals.

Accordingly, the present study investigated the anatomical correlates of loneliness in WM structures in healthy young people. We hypothesised that the structural white matter correlates of loneliness would be associated with empathy and self-efficacy. We tested this hypothesis by performing multiple regression analysis on loneliness scores. We used VBM to assess rWMD and FA on diffusion tensor imaging (DTI) to identify the neural correlates of loneliness in healthy subjects. Additionally, although women feel more empathy than men, and although the neural networks supporting empathy are modulated by gender^19^, evidence of gender differences in loneliness remains controversial^20^. Hence, we also investigated the existence of such gender differences.

## Results

### Behavioural data

**Figure 1:**
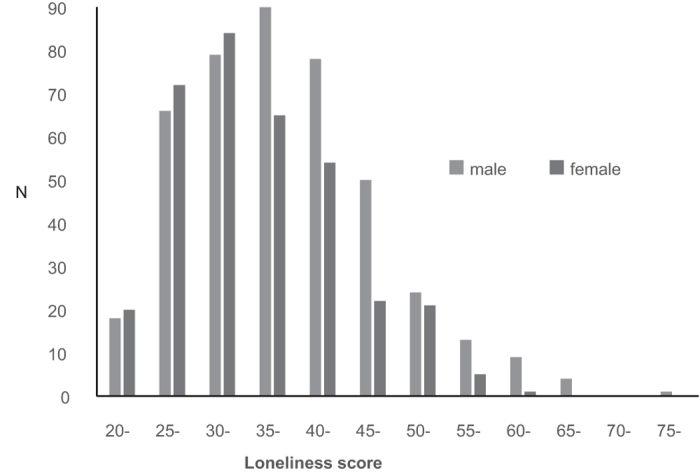
Distribution of Loneliness Scale scores in males and females. Histogram showing the distribution of loneliness scores for all subjects.

Figure 1 shows the distribution of the UCLA Loneliness Scale score in males and females. Table 1 shows that scores on the UCLA Loneliness Scale were significantly negatively correlated with those on the EQ (*r* = −0.405, *P* [uncorrected] < 0.0001, *P* [two-tailed corrected using the Bonferroni method] < 0.001) and General Self-Efficacy Scale (GSES) (*r* = −0.543, *P* [uncorrected] < 0.0001, *P* [two-tailed corrected using the Bonferroni method] < 0.001), whereas EQ scores were significantly positively correlated with GSES scores (*r* = 0.510, *P* [uncorrected] < 0.0001, *P* [two-tailed corrected using the Bonferroni method] < 0.001). Even when we conducted correlation analyses between loneliness, EQ, and GSES controlled for gender, age, and RAPM scores, we found significant correlations between loneliness and EQ (*r* = −0.371, *P* [uncorrected] < 0.0001, *P* [two-tailed, corrected using the Bonferroni method] < 0.001) and between loneliness and GSES (*r* = −0.534, *P* [uncorrected] < 0.0001, *P* [two-tailed, corrected using the Bonferroni method] < 0.001).

Table 2 shows the age and the Raven’s Advanced Progressive Matrix (RAPM), UCLA Loneliness Scale, EQ and GSES scores. The loneliness (*P* < 0.01) scores were significantly higher for males than females using one-way analysis of variance (ANOVA).

### MRI data

#### Loneliness scores

**Figure 2:**
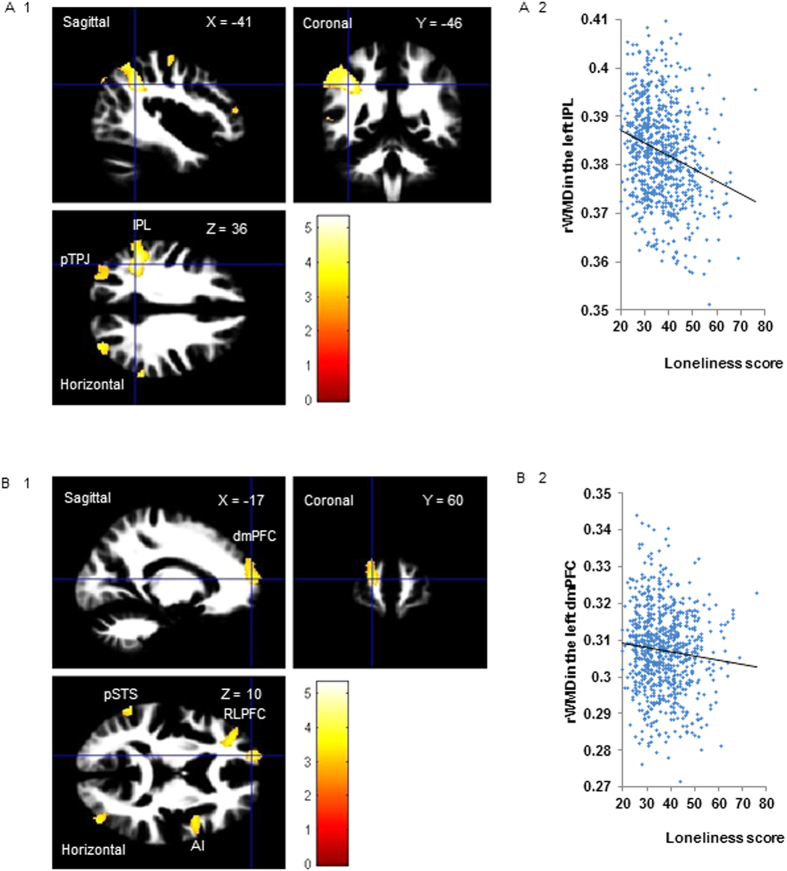
Regions showing a correlation between rWMD and UCLA Loneliness Scale scores. The red-to-yellow colour scale indicates the *t*-score for the negative correlation between rWMD and UCLA Loneliness Scale score (*P* < 0.0025, uncorrected and k > 100 for visualisation purposes). Regions showing correlations were overlaid on preprocessed, smoothed-mean rWMD images using templates created from 63 subjects who participated in a previous experiment in our laboratory. The left inferior parietal lobule (IPL, x, y, z = −41, −46, 36) showed areas of significant correlation in the bilateral inferior parietal lobule (IPL) and left posterior temporoparietal junction (pTPJ) (a1). A scatterplot of UCLA Loneliness Scale scores and mean rWMD values in the significant clusters in the left IPL (a2). Dorsomedial prefrontal cortex (dmPFC, x, y, z = −17, 60, 10) showing regions of significant correlation in the left posterior superior temporal sulcus (pSTS), right anterior insula (AI), left dmPFC, and left rostrolateral prefrontal cortex (RLPFC) (b1). A scatterplot of the UCLA Loneliness Scale scores and mean rWMD values in the significant clusters in the left dmPFC (b2).

We investigated the relationship between rWMD and individual differences on the UCLA Loneliness Scale. A multiple regression analysis of age, gender, general intelligence, and total intracranial volume (TIV: total GM volume + total WM volume + total cerebrospinal fluid volume) revealed that the loneliness score was significantly negatively correlated with rWMD in the bilateral IPL, left pSTS, left posterior TPJ, right AI, left mPFC, and left rostrolateral prefrontal cortex (RLPFC) at *P* < 0.05 (false discovery rate [FDR] corrected) (Fig. 2 and Table 3). We found no significant positive correlations between rWMD and UCLA Loneliness Scale score at *P* < 0.05 (FDR corrected).

The ANCOVA revealed no significant interaction effect between loneliness scores and gender on rWMD at *P* < 0.05 (FDR corrected).

We investigated FA related to individual differences in scores on the UCLA Loneliness Scale. The number of b = 0 images (1 or 3) was included in the model because the number of b = 0 images affects the estimation of FA values^21^. A multiple regression analysis including age, gender, and general intelligence revealed no significant effect at *P* < 0.05 (FDR corrected). The ANCOVA revealed no significant interaction between loneliness scores and gender on FA (*P* < 0.05 FDR corrected) on a whole-brain level.

#### Associations between rWMD and correlates of the EQ and GSES

We performed multiple regression analysis on loneliness scores, treating sex, age, RAPM score, and TIV as covariates, with regions of interest (ROIs) determined by whole-brain analysis being related to EQ or GSES scores; statistical significance was set at *P* < 0.05 (uncorrected) (Table 3). The rWMDs (cluster size of more than 100 voxels) in the right IPL (peak voxel of MNI: x = 41, y = −76, z = 19), the left IPL, the right AI, and the left pTPJ overlapped significantly with both EQ and GSES scores. The rWMDs (cluster size of more than 100 voxels) in the left pSTS overlapped significantly with EQ scores, whereas those in the dmPFC overlapped with GSES scores.

**Figure 3:**
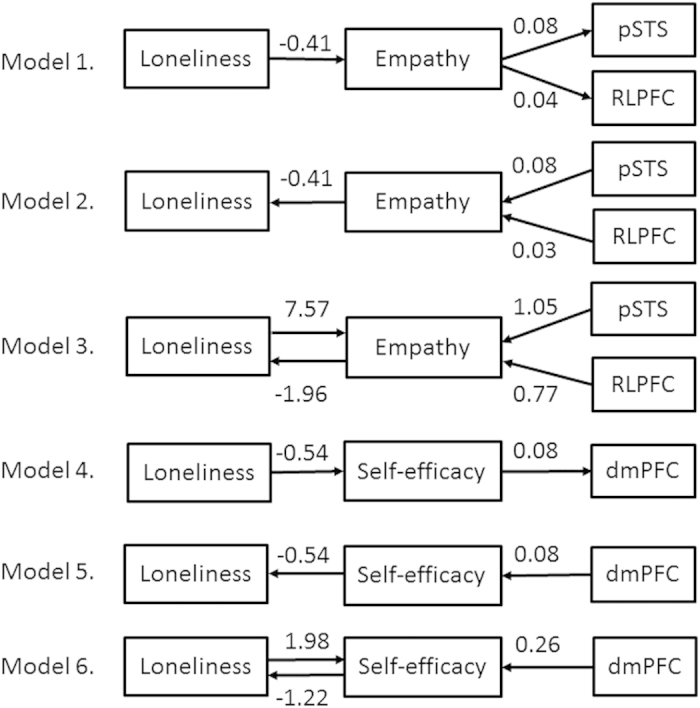
Model 1: Loneliness reduced rWMD in the pSTS and RLPFC via empathy. Model 2: Reduced rWMD in the pSTS and RLPFC with increased loneliness via empathy. Model 3: Modified Model 3, with interaction between loneliness and empathy. Model 4: Loneliness reduced rWMD in the dmPFC via self-efficacy. Model 5: Reduced rWMD in the dmPFC with increased loneliness via self-efficacy. Model 6: Modified Model 5 with an interaction between loneliness and self-efficacy. One-headed arrows indicate the direction of the observed regression. The numbers on the arrows represent standardised regression coefficients. Error components are omitted for simplicity.

Figure 3 shows the link between loneliness and mean rWMD values within the specific significant clusters identified through the aforementioned analyses mediated by individual differences in empathy or self-efficacy. Among Models 1, 2, and 3, Model 3 provided the best fit (goodness of fit [GFI] = 0.997, adjusted goodness of fit [AGFI] = 0.984, comparative fit index [CFI] = 0.980, and root mean square error of approximation [RMSEA] = 0.045). The results for the other two models were as follows: GFI = 0.986, AGFI = 0.953, CFI = 0.887, RMSEA = 0.088 for Model 1; and GFI = 0.986, AGFI = 0.952, CFI = 0.885, RMSEA = 0.089 for Model 2. Among Models 4, 5, and 6, Models 4 and 5 provided the best fit (GFI = 0.998, AGFI = 0.985, CFI = 0.993, and RMSEA = 0.049). The results for Model 6 were as follows: GFI = 1, CFI = 1, RMSEA = 0.344.

## Discussion

To our knowledge, the present study is the first to reveal a correlation between rWMD and loneliness and to show that empathy and self-efficacy are important factors with respect to loneliness^6^. We found that the UCLA Loneliness Scale score was significantly negatively correlated with rWMD in the bilateral IPL, right AI, left pSTS, pTPJ, dmPFC, and RLPFC in both males and females. Moreover, we found significant overlap between scores on the UCLA Loneliness Scale and inverse scores on the EQ with respect to wide rWMDs (cluster size of more than 100 voxels) in the bilateral IPL, the right AI, the left pSTS, the pTPJ, and the RLPFC, and between GSES scores and inverse scores on the GSES with respect to wide rWMDs in the bilateral IPL, the right AI, the left pTPJ, and the dmPFC. Furthermore, we found a significant negative correlation between the UCLA Loneliness Scale scores and the EQ and GSES scores. These results are consistent with our hypothesis that the neural correlates of loneliness are related to empathy and self-efficacy, suggesting that changes in WM structure reflect dysfunction, because reduced rWMD indicates less myelination in the region considered, which would delay the neural transmission in the area and in the neural network.

First, loneliness empathy, and self-efficacy were widely associated with the bilateral IPL and the right AI and left pTPJ, which are involved in both self-cognition and social cognition. The IPL plays an integral role in social processes such as empathy^22^. Additionally, the mirror neuron system, which facilitates understanding of others’ intentions and may be a neural substrate of empathy, is found in the IPL^23^. In particular, the right IPL is believed to play an important role in ascribing intentions to others^10^. The AI is associated with empathy for pain^24^. The AI may subserve neural representations of feeling and bodily states in the self and may play a crucial role in the emergence of emotions related to social interactions^25^. Interestingly, a lesion in the AI interferes with an individual’s ability to think of themselves as or feel romantically connected to others, even though the lesion does not interfere with the individual’s ability to feel sexual desire for others^26^. Moreover, the AI plays a pivotal role in self-efficacy related to emotions such as pain and disgust, whether they are experienced personally or observed in others^11^, and in internal states affected by self-referential processing, such as social exclusion^27^. From another point of view, mutual gaze contacts are processed by direct fibre pathways between the AI and pSTS. Decoding gaze motion is critical for predicting the intentions, future actions, and attitudes of other people as well as for the appraisal of self-relevance^28^. The TPJ plays a central role in various aspects of social cognition, such as theory of mind and empathy^29^. In particular, the TPJ controls representations of the self or of other individuals across a variety of sociocognitive processes, both at a low level (agency discrimination, visual perspective taking, control of imitation) and at a higher level (mentalising, empathy)^30^. Consequently, people with dysfunctions in these regions have difficulty feeling empathy for those in need and also lack the self-efficacy to support them, which is necessary for the formation of close relationships. They are therefore less aware of the subjective feelings of pain, distress, and social exclusion that underlie loneliness.

Second, loneliness and empathy were widely associated with white matter changes underlying the left pSTS and left RLPFC—regions that play important roles in cognitive functioning in socially oriented domains. The pSTS is activated by changeable or dynamic facial characteristics, such as gaze, facial expression, and lip movements^31^. For example, the recruitment of multimodal sensory regions in the pSTS indicates detection of gaze direction via complex visual analysis^32^. A previous study on loneliness showed that GM volume in the pSTS was negatively correlated with individual differences in loneliness and social-perception skills^8^. We previously suggested that the principal function of the STS may be to analyse changing sequences of auditory or visual inputs and interpret their communicative significance. Through this function, the STS plays a key role in the perception of social signals, language, and speech^10^. Thus, a dysfunctional pSTS may disrupt visual and auditory communication, reducing cognitive empathy and increasing feelings of loneliness. The left RLPFC assists with verbal or semantic relational integration as needed and is engaged only when people need to consider the higher-order relationship between two individual relations^33^. A previous study showed that RLPFC activation increased in adults when relational integration was required^34^. Furthermore, the left RLPFC plays a central role in social reasoning^35^. Accordingly, a deficit in RLPFC function would disrupt higher cognitive empathy with regard to verbal or semantic relational integration, comprehension of higher-order interactions, and social reasoning, all of which are key to forming stable relationships.

Third, we should examine possible mediating mechanisms that can explain how lower empathy can increase loneliness, whereas increased loneliness can increase empathy. We do so based on the best-fit model, Model 3. We can understand the former mechanism relatively easily because dysfunction of pSTS and RLPFC related to cognitive empathy could lead to feelings of loneliness, based on the aforementioned mechanism. However, some might be sceptical about the latter mechanism. From an ontological perspective, loneliness could be described as having diametrical symptoms as a dysfunction and as a meaningful function for existence^36^. In fact, empathy scores correlated positively with loneliness, which was experienced positively in one psychological study^37^. The primary way to overcome loneliness is to improve one’s social relations^1^. Individuals who feel loneliness accompanied by distressing anxiety might be prompted to consider how to improve their relations with other people. They might then endeavour to better understand others’ thoughts and wishes. Consequently, they might improve their ability to feel empathy.

Fourth, loneliness and self-efficacy were widely associated with the mPFC, which plays an important role in cognitive functions related to the self. According to D’Argembeau *et al.*^38^, cortical midline structures appear to play a key role in self-referential processing; notably, the neural correlates of self-referential processing are most consistently observed in the mPFC. A previous fMRI study found that the mPFC was engaged during the processing of positive images, whereas the lateral PFC responded differentially to negative items^39^. Because MRI data revealed activity in the dorsal rather than ventral region of the mPFC, we focused on dmPFC function. The dmPFC activity is associated with self-referential processing, such as self-referential criticism in social situations^40^, rather than with processing related to other individuals; thus, dmPFC activation may support the retrieval, evaluation, and/or integration of self-related information to construct a coherent self-image^41^. Accordingly, dmPFC dysfunction would interfere with the maintenance of self-efficacy through self-referential processing. Interestingly, a study involving mice has shown that social isolation leads to permanent reductions of myelination in the PFC in mice isolated for two weeks immediately after weaning, whereas these reductions in adult mice are reversible. The latter finding seems to raise the possibility that loneliness may be a cause as well as a consequence of changes in rWMD^42^. The structural integrity of white matter strongly affected the capacity for emotional empathy^43^, which plays a critical role in human communication^44^. A lower capacity for empathy appears to lead to loneliness.

As we have previously noted^9^, regions with significant results in the rWMD analyses were not identified on FA (structural integrity), suggesting that these functions have different physiological bases. This supposition is supported by previous reports of disparities in the distribution of associations between FA and group/individual differences on the one hand and those of rWMD and group/individual differences on the other^45^. This finding is not surprising, considering that FA and rWMD are moderately to weakly related^46^ and that associations between the two appear to be particularly weak in deep WM areas^45^. Furthermore, our FA analyses focused on areas that were most likely to be WM. FA and rWMD are believed to be highly sensitive to the different characteristics of WM^46^. As we have previously noted^10^, myelination, myelin membrane thickness, and increases in axon diameter are associated with increases in FA, rWMD, and cognitive ability. Other factors that may selectively affect rWMD and leave diffusion anisotropy relatively unchanged include the number and size of glial cells (which constitute a major portion of WM), the number of axon collateral spines, and a pure increase in WMD (or an increase in all of the physiological components of WM while the composites remain the same), resulting in the present significant associations specifically observed in rWMD.

We should also explain why there was a negative correlation between UCLA Loneliness Scale scores and EQ scores. This negative correlation could be due to negative perceptions held by lonely individuals about personal social proclivities, leading to difficulty in understanding the mental states of others (i.e., a decrease in empathy)^4^. Although empathy involves multidimensional constructs—including mentalising, affective communication, and social attachment^47^—one of the most important constructs may be the ability to understand the intentions, feelings, and emotions of others^4^.

Our study has some limitations. Firstly, this study was cross-sectional, and therefore it could not determine the direction of causality among the factors. We defined ROIs according to EQ or GSES scores, with statistical significance set at *P* < 0.05, uncorrected (i.e., we did not apply a correction). Longitudinal cross-lag structural- equation analyses and experimental studies in humans have shown loneliness to affect (and be affected by) social cognition and behaviour. For instance, there is now a sizable literature consistent with the notion that loneliness in humans (as well as social isolation from preferred partners in animal studies) triggers a behavioural repertoire of self-preservation 7. Given that empathy has been shown to vary as a function of the perceived connection to the observed individual^48^, and given that loneliness reflects perceived social isolation (i.e., disconnection)^49^, it is not surprising that loneliness can lead to diminished empathy. Similarly, loneliness can lead to a decrease in perceived control, just as a decrease in control over one’s relationships can increase feelings of loneliness. Thus, it appears as though loneliness can influence and be influenced by these related social processes. Further studies using larger and more representative samples are needed to determine whether our results are generalisable across a wider range of populations. For more details, see the Supplementary Discussion section.

In conclusion, the results of this study suggest that the regions related to loneliness may overlap with the right IPL, AI, and pTPJ (in terms of both empathy and self-efficacy); the left pSTS and RLPFC (mainly in terms of empathy); and the left IPL and dmPFC (mainly in terms of self-efficacy). Among the unique findings of this study are the finding that the RLPFC seems to play an important role in loneliness insofar as empathy (in terms of verbal or semantic relational integration) is involved. Similarly, the dmPFC may play a critical role in loneliness, insofar as self-efficacy (in terms of self-referential processing, such as retrieval, evaluation, criticism, and integration of self-related information) is involved. Our study identified some changes in white matter that may provide insights into the underlying mechanisms associated with loneliness. These insights may help to mitigate certain issues related to feelings of loneliness, particularly in young people.

## Methods

### Subjects

We enrolled 776 healthy right-handed individuals (432 males and 344 females) in the present study as part of our ongoing project to investigate the associations among brain imaging. The subjects comprised cognitive students aged 18–27 years (mean ± SD, 20.2 ± 1.5) from Tohoku University in Japan. Written informed consent was obtained from all subjects for the project in which they participated, in accordance with the Declaration of Helsinki (1991). Our study was approved by the ethics committee of Tohoku University Graduate School of Medicine. For more details, see the Supplementary Methods section.

### Psychological outcome measures

#### Assessment of loneliness

The University of California-Los Angeles (UCLA) Loneliness Scale (Version 3) is highly reliable and is the most widely used questionnaire on loneliness^50^. The questionnaire has high internal consistency (coefficient alpha = 0.89–0.94) and a test–retest reliability of 0.73 over a two-month period. Convergent validity for the scale was confirmed by significant correlations with other measures of loneliness^50^. We used the Japanese version of the UCLA Loneliness Scale, whose reliability and validity have been reported to be acceptable^51^.

The UCLA Loneliness Scale consists of 20 items that assess perceptions of social isolation and loneliness. Participants rate each item on a scale from 1 (Never) to 4 (Often). Higher scores reflect greater loneliness. Examples of the questions include “How often do you feel that you lack companionship?” and “How often do you feel that no one really knows you well?” For more details, see the Supplementary Methods section.

#### Assessment of psychometric measures of general intelligence

General intelligence was measured using RAPM, which is widely considered to be the best measure of general intelligence, adjusting for the effect of individual psychometric measures of general intelligence on brain structures^52^. The test contains 36 nonverbal items requiring fluid reasoning ability. The test score (the number of correct answers in 30 min) was used as a psychometric index of individual intelligence. For more details, see the Supplementary Methods section.

#### Assessment of empathy

A Japanese version of the EQ questionnaire^53^ was administered, and the score was used as an index of empathy. The test consists of 40 EQ items and 20 filler items that are not scored. The scale consists of self-descriptive statements scored on a four-point scale, ranging from “Strongly Disagree” to “Strongly Agree”. Half of the items are worded to produce an “agree” response, with the rest being worded to produce a “disagree” response. The items were randomised to control for response bias. Each strong empathic response was awarded two points, while each slightly empathic response was awarded one point (i.e., each item was scored as 2, 1, or 0), resulting in a range of total scores from 0 to 80 points.

#### Assessment of general self-efficacy

The GSES, developed by Sherer and Adams^54^, measures an individual’s general sense of self-efficacy for events that occur in various everyday settings. Self-efficacy was nurtured by the individuals themselves, based on phenomena such as vicarious experiences and emotional arousal^5^. General self-efficacy reflects an overarching pattern of judgement regarding one’s own efficacy across various domains of functioning^12^ and measures confidence in one’s general capacity to handle tasks^55^. The validity of the GSES for the variables under study has been found to be consistent across countries and subjects. Thus, this psychometric scale appears to tap into a universal construct and yields meaningful relationships with other psychological constructs^12^. We used the Japanese version of the GSES, which has good reliability and validity^56^. The scale included a questionnaire with 23 items using a five-point Likert scale, with total scores ranging from 23 to 115 and higher scores indicating a greater sense of self-efficacy. For more details, see the Supplementary Methods section.

#### Behavioural data analysis

The behavioural data were analysed using the Statistical Package for the Social Sciences version 16.0 (SPSS, Inc., Chicago, IL, USA). Relationships among the psychological measures (RAPM, UCLA Loneliness Scale, EQ and GSES) and additional covariates (age, gender) were assessed using one-way ANOVA. We used the Pearson correlation coefficient to test for correlations among age, RAPM, UCLA Loneliness Scale, EQ, and GSES scores. *P*-values <0.05, two-tailed corrected using the Bonferroni method were deemed statistically significant.

#### Image acquisition

All MRI data were acquired using a 3-T Philips Intera Achieva scanner. High-resolution T1-weighted structural images (T1WIs; 240 × 240 matrix, TR = 6.5 ms, TE = 3 ms, FOV = 24 cm, slices = 162, slice thickness = 1.0 mm) were collected using a magnetisation-prepared rapid gradient echo sequence.

Diffusion-weighted data were acquired using a spin-echo EPI sequence (TR = 10,293 ms, TE = 55 ms, Δ = 26.3 ms, δ = 12.2 ms, FOV = 22.4 cm, 2 × 2 × 2 mm^3^ voxels, 60 slices, SENSE reduction factor = 2, number of acquisitions = 1). For more details, see the Supplementary Methods section.

#### Preprocessing of T1-weighted structural data

Structural data were preprocessed using Statistical Parametric Mapping software (SPM8; Wellcome Department of Cognitive Neurology, London, UK) implemented in Matlab (Mathworks, Inc., Natick, MA, USA). Next, using this existing template, the DARTEL procedure was performed on all subjects in the present study using the default parameter setting. The resultant images were spatially normalised to the Montreal Neurological Institute (MNI) space to produce images of 1.5 × 1.5 × 1.5 mm^3^ voxels. Subsequently, all normalized rWMD images were smoothed by convolving them with a 12-mm full-width at half-maximum (FWHM) isotropic Gaussian kernel. We used these rWMD images derived from the WM segments of the structural images. For more details, see the Supplementary Methods section.

#### Preprocessing diffusion imaging data

In DTI, the FA in each voxel is a measure of the degree of diffusion anisotropy. FA reflects the angle (degree of directionality) of cellular structures within fibre tracts and is thus an indicator of fibre integrity^57^. For more details, see the Supplementary Methods section.

#### Statistical analysis of rWMD

Whole-brain multiple regression analysis was used to assess the relationship between rWMD and UCLA Loneliness Scale scores. The covariates included gender, age, RAPM score, and total intracranial brain volume (TIV). For each covariate, the “overall mean” was used for mean centring. Statistical significance was set at *P* < 0.05 (FDR corrected)^58^. For more details, see the Supplementary Methods section.

#### Associations between rWMD and psychological correlates of the EQ and GSES

To elucidate the relationship between loneliness and EQ and GSES scores, we also used imaging data to verify whether there was significant statistical overlap in the aforementioned regions. That is, we first established ROIs according to EQ or GSES scores at *P* < 0.05, uncorrected. Then, we performed multiple regression analysis on loneliness scores, treating sex, age, RAPM scores, and TIV as covariates with the ROIs. Statistical significance was set at *P* < 0.05 (FDR corrected).

Structural equation modelling (SEM) is useful for assessing mediation because it offers several interesting alternative ways to explore mediation effects^59^. Hence, we conducted SEM to demonstrate that the link between loneliness and mean rWMD values within the specific significant clusters identified through the aforementioned analyses was mediated by individual differences in empathy or self-efficacy. All of the factors that made significant independent contributions to scores on the UCLA Loneliness Scale were entered into linear structural equation systems (AMOS 18) in order to explore the interrelationships of the scores related to EQ, GSES, and the UCLA Loneliness Scale. We constructed a model (Model 1) in which loneliness reduced the rWMD of the left pSTS and RLPFC, mediated by empathy (EQ). We also constructed the inverse model (Model 2), in which reduction of the rWMD of the left pSTS and RLPFC increased loneliness, again mediated by EQ. Then, after selecting the model with the better fit, we constructed an additional model that reflected bidirectional effects between loneliness and EQ (Model 3). We constructed another model (Model 4), in which loneliness reduced the rWMD of the left dmPFC, mediated by self-efficacy (GSES). We also created the inverse model (Model 5), in which reducing the rWMD of the left pSTS and RLPFC increased loneliness, mediated by GSES. Then, after selecting the model with the better fit (between Models 4 and 5), we constructed a model reflecting bidirectional effects between loneliness and GSES (Model 6).

#### Statistical analysis of FA

Statistical analyses relating to FA were performed using SPM8. We investigated the relationship between FA and UCLA Loneliness Scale scores in all subjects. The number of b = 0 images (1 or 3) was included in the model because this value affects the estimation of FA values^60^. A common effect of the number of b = 0 images on FA values was assumed for both genders. The areas used in the FA analyses were confined to those with WMC values >0.99 in the average normalised WMC image.

Furthermore, we investigated gender differences in UCLA Loneliness Scale scores using the same methods as those used in the statistical analysis of rWMD, except that in the models used in FA analyses, TIV was replaced by the number of b = 0 images in each analysis. Statistical significance was set at *P* < 0.05 (FDR corrected).

## Additional Information

**How to cite this article**: Nakagawa, S. *et al.* White matter structures associated with loneliness in young adults. *Sci. Rep.* **5**, 17001; doi: 10.1038/srep17001 (2015).

## Supplementary Material

Supplementary Discussion and Methods

## Figures and Tables

**Table 1:** Pearson correlations among age, scores on RAPM, UCLA Loneliness Scale, EQ, and GSES.

	Age	RAPM	Loneliness	EQ	GSES
Age	–				
RAPM	−0.017	–			
Loneliness	−0.102*	0.045	–		
EQ	−0.160^**^	−0.075	−0.405^**^	–	
GSES	−0.211	−0.061	−0.543^**^	0.510^**^	–

Abbreviations: EQ Empathising Quotient; GSES, General Self-Efficacy; Loneliness, UCLA Loneliness Scale; RAPM, Raven’s Advanced Progressive Matrix.

^*^P < 0.05, **P < 0.001 (two-tailed corrected using the Bonferroni method)

**Table 2:** Sex differences in age, scores on RAPM, UCLA Loneliness Scale, EQ, and GSES, and one-way ANOVA results.

	Total		Males (*N* = 432)		Females (*N* = 344)		*P*	*F*
Measure	Mean	SD	Mean	SD	Mean	SD		
Age	20.7	1.8	20.8	2.0	20.6	1.7	0.089	2.9
RAPM	28.6	3.7	28.9	3.8	28.3	3.7	0.018*	5.6
Loneliness	37.0	9.2	38.3	9.6	35.4	8.3	<0.001**	19.8
EQ	31.2	10.1	29.1	9.7	33.8	9.9	<0.001**	43.4
GSES	69.7	12.4	69.3	13.0	70.3	11.6	0.27	1.2

Abbreviations: ANOVA, analysis of variance; EQ, empathy quotient; GSES, generalized self-efficacy scale; Loneliness, UCLA Loneliness Scale; RAPM, Raven’s Advanced Progressive Matrix; SD, standard deviation.

*P < 0.05, **P < 0.001.

**Table 3:** Brain regions showing a significant correlations between rWMD and scores on the UCLA Loneliness Scale, EQ and GSES scores.

								Using a mask related to EQ				Using a mask related to GSES			
Brain region	R/L	x	y	z	*t* score	Corrected *P* (FDR)	Cluster size (k_E_)	*t* score	Corrected *P* (FDR)	Clustersize(k_E_)	*β*	*t* score	Corrected *P*(FDR)	Cluster size (k_E_)	*β*
IPL	R	41	−72	18	4.51	0.011^*^	238	4.51	0.001^**^	467	0.077	4.51	0.001^**^	660	0.122
	R	62	-42	37	3.73	0.046^*^	3	3.73	0.005^**^	109	0.108	3.74	0.003^**^	68	0.075
	L	−41	−46	36	5.33	0.004^**^	860	5.33	<0.001^***^	849	0.096	5.34	<0.001^***^	1534	0.145
pSTS	L	−62	−52	9	3.99	0.031^*^	15	3.91	0.004^**^	182	0.082	4.00	0.002^**^	52	0.044
AI	R	45	8	9	3.79	0.042^*^	2	3.79	0.005^**^	396	0.094	3.79	0.003^**^	398	0.123
pTPJ	L	−30	−79	40	3.81	0.041^*^	11	3.81	0.005^**^	253	0.098	3.81	0.003^**^	293	0.1
dmPFC	L	−17	60	10	4.02	0.029^*^	35	4.03	0.003^**^	89	0.061	4.03	0.002^**^	545	0.097
RLPFC	L	−29	41	9	3.77	0.043^*^	5	3.77	0.005^**^	247	0.073	3.75	0.003^**^	49	0.048

^*^P  < 0.05, ^**^P < 0.01, ^***^P < 0.001 Abbreviations: AI, anterior insula; dmPFC, dorsomedial prefrontal cortex; EQ, empathy quotient; GSES, generalized self-efficacy scale; FDR, false discovery rate; IPL, inferior parietal lobule; L, left; pSTS, post superior temporal sulcus; pTPJ, posterior tempo-parietal junction; R, right; ROIs, regions of interest; rWMD, regional white-matter density; RLPFC, rostro lateral prefrontal cortex.
