# Supplementary material for: White matter structures associated with loneliness in young adults
Source: Sci Rep. 2015 Nov 20;5:17001. doi: 10.1038/srep17001 (PMC4653806; doi:10.1038/srep17001)
Supplement: Supplementary Discussion and Methods [file srep17001-s1.doc]

**White matter structures associated with loneliness in young adults**

Seishu Nakagawa^a,b,*^, Hikaru Takeuchi ^c^, Yasuyuki Taki^c,d,e^, Rui Nouchi^f,g^, Atsushi Sekiguchi^b,d^, Yuka Kotozaki^g^, Carlos Makoto Miyauchi^b,h^, Kunio Iizuka^b,I^, Ryoichi Yokoyama^b,j^, Takamitsu Shinada^b^, Yuki Yamamoto^b^, Sugiko Hanawa^b^, Tsuyoshi Araki^g^, Hiroshi Hashizume^c^, Keiko Kunitoki^k^, Yuko Sassa^c^, Ryuta Kawashima^b,c,g^

^a^ Department of Psychiatry, Tohoku Pharmaceutical University

^b^ *Department of Functional Brain Imaging, Institute of Development, Aging and Cancer, Tohoku University, Sendai, Japan*

^c^ *Division of Developmental Cognitive Neuroscience, Institute of Development, Aging and Cancer, Tohoku University, Sendai, Japan*

^d^ *Division of Medical Neuroimaging Analysis, Department of Community Medical Supports, Tohoku Medical Megabank Organization, Tohoku University, Sendai, Japan*

^e^ *Department of Nuclear Medicine and Radiology, Institute of Development, Aging and Cancer, Tohoku University, Sendai, Japan*

^f^ *Human and Social Response Research Division, International Research Institute of Disaster Science, Tohoku University, Sendai, Japan*

^g^ *Smart Ageing International Research Center, Institute of Development, Ageing and Cancer, Tohoku University, Sendai, Japan*

^h^ *Graduate School of Arts and Sciences, Department of General Systems Studies, The University of Tokyo*

^i^ *Department of Psychiatry, Tohoku University Graduate School of Medicine, Sendai, Japan*

^j^ *Japan Society for the Promotion of Science, Tokyo, Japan*

**Supplementary Discussion**

Our study has some limitations. The subjects were healthy, young, highly educated college students; thus, our findings may not be generalisable to populations with a different educational background. This limitation is inherent in investigations using cohorts of college students^1,2^. Furthermore, our study was limited by selection bias because all of the subjects were young; however, given the high prevalence of loneliness in young people^3^, it is important to focus on the younger generation.

**Supplementary Methods**

**Subjects**

All subjects had normal vision, and none had a history of neurological or psychiatric illness. Handedness was evaluated using the Edinburgh Handedness Inventory^4^ functions, ageing, genetics, and daily habits^5^. Additional psychological tests and magnetic resonance imaging (MRI) scans were performed but are not included in this report.

**Psychological outcome measures**

**Assessment of loneliness**

In response to concerns about the way in which negative wording of the items may have affected scores, the third version of the scale included 10 items worded in a negative or lonely direction and 10 items worded in a positive or non-lonely direction. Responses to the 10 items worded in a positive or non-lonely direction were reversed (i.e., 1 = 4, 2 = 3, 3 = 2, 4 = 1) before scoring^6^. A significant relationship was found between the loneliness score and the number of close friends (*r* = -0.27) ^6^.

**Assessment of psychometric measures of general intelligence**

Additionally, the RAPM was used to exclude the possibility of a significant correlation between WM structure and the UCLA Loneliness Scale resulting from a relationship between general intelligence and the Loneliness Scale or WM structure.

**Assessment of general self-efficacy**

Several conceptual relationships have been found between the GSES and other personality constructs (e.g., personal control, social desirability, ego strength, interpersonal competence, and self-esteem) as well as vocational, educational, and military success^7^. Belief in one’s ability to exert control in relationships may lead to greater social participation and decreased loneliness^8^. Loneliness may reflect a discrepancy between an individual’s desired level of social participation and his/her actual social network^8^.

**Image acquisition**

The diffusion weighting was isotropically distributed along 32 directions (b value = 1000 s/mm^2^). Additionally, three images with no diffusion weighting (b value = 0 s/mm^2^; b = 0 images) and one b = 0 image were acquired from 440 and 127 subjects, respectively, using a spin-echo EPI sequence (TR = 10,293 ms, TE = 55 ms, FOV = 22.4 cm, 2 × 2 × 2 mm^3^ voxels, 60 slices). From the collected images, FA maps and apparent diffusion coefficient (ADC) maps were calculated using the commercially available diffusion tensor analysis package on the MR consol. Calculations were performed using a previously reported method^9^.

**Preprocessing of T1-weighted structural data**

T1-weighted structural images of each individual were segmented into six tissue types using the new segmentation algorithm in SPM8. In this process, the grey matter tissue probability map (TPM) was manipulated from maps implemented in the software so that the signal intensities of voxels (tissue probability of the default tissue grey matter [TPM] + white matter tissue probability of the default TPM) >0.25 became 0. When the manipulated grey matter TPM was used, dura mater was less likely to be classified as grey matter (compared with the default grey matter TPM), without other substantial segmentation problems. The new segmentation process used default parameters, except that affine regularisation used the International Consortium for Brain Mapping (ICBM) template for East Asian brains. We then used the diffeomorphic anatomical registration through exponentiated lie algebra (DARTEL) registration process in SPM8 to import images of five grey matter TPMs resulting from the new segmentation process. First, the template for the DARTEL procedure was created using the imaging data from 63 subjects who participated in a previous experiment in our laboratory^10^ and were also included in the present study.

**Preprocessing diffusion imaging data**

A post-mortem pathology study showed that the FA value is highly correlated with the amount of myelin. Cognitive processing speed, which is thought to be associated with the structure of WM pathways^11^, is positively correlated with FA in various regions^11^. Moreover, FA is correlated with cognitive abilities related to social and emotional competences. FA varies between 0, representing isotropic diffusion, and 1, indicating diffusion along one plane or direction. The FA images were preprocessed using the SPM8 software implemented in Matlab (Mathworks, Inc.). Using the new segmentation algorithm implemented in SPM8, FA images of each individual were segmented into six tissues (first new segmentation). Default parameters were used in this process, except that affine regularisation used the ICBM template for East Asian brains, and the sampling distance (the approximate distance between sampled points when estimating the model parameters) was 2 mm. We next synthesised the FA image and ADC map. In this synthesised image, the areas with a WM tissue probability of >0.5 were the FA image multiplied by −1 (hence, this synthesised image showed a very clear contrast between the WM and the other tissues); the remaining area was the ADC map (the reasons for this procedure are explained below). The synthesised image from each individual was then segmented using the new segmentation algorithm in SPM8, employing the same parameters described above (second new segmentation). We used this two-step segmentation process because the FA image had a relatively clear contrast between GM and WM and also between WM and CSF, and because the first new segmentation step can distinguish WM tissue from other tissues. In contrast, the ADC map showed a clear contrast between GM and CSF, and the second new segmentation could separate GM. As the ADC map alone did not show a clear contrast between WM and GM, it was necessary to use a synthesised image along with the two-step segmentation process.

We then proceeded to the DARTEL registration process implemented in SPM8. In this process, we used the DARTEL import image of the GM TPM produced in the second new segmentation process as the GM input for the DARTEL process. The WM input for the DARTEL process was created as follows: First, the raw FA image was multiplied by the WM TPM from the second new segmentation process within the areas with a WM probability >0.5 (the signals from other areas were set to 0). Then, this FA image × the WM TPM was coregistered and resliced to the DARTEL import WM tissue probability image from the second segmentation. The template for the DARTEL procedure was created using imaging data from 63 subjects who participated in a previous experiment in our laboratory^10^ and who were also included in the present study (these subjects had the same characteristics as the participants in the present study). We created the DARTEL template using images from a subset of subjects. Because our study is ongoing, and given finite time and resources, it is not feasible to reprocess the images every time new subjects are added to the study. Furthermore, 63 is a relatively large sample size—larger than that used in previous studies and thus not problematic in terms of statistical power. Using the existing template, the DARTEL procedure was then performed on the subjects in the present study. The parameters of the procedure were changed to improve the accuracy as follows. The number of Gauss–Newton iterations to be performed in each outer iteration was set to 10, and in each outer iteration, we used an eightfold increase in time points (relative to the default values) to solve the partial differential equations. The full multi-grid matrix solver was set to eight cycles, and eight relaxation iterations were performed in each multi-grid cycle. The resultant synthesised images were spatially normalised to the MNI space. Using the parameters for these procedures, the raw FA map, the GM segmentation map (GM concentration map), and the WM segmentation map (WM concentration [WMC] map) from the second new segmentation process were normalised to give 1.5 × 1.5 × 1.5-mm images three voxels. The FA image × the WM TPM was used in the DARTEL procedure because this image included different signal intensities within the WM tissues. The normalisation procedure used the intensity differences to adjust the image to the template from the perspective of the outer edge of the tissue and within the WM tissue. No modulation was performed in this normalisation procedure.

We then created an average image based on the normalised WM segmentation images from the 63 subjects and the created mask image consisting of voxels with a WM signal intensity >0.99. We applied this mask image to the normalised FA image. Finally, the masked normalised FA image was smoothed by convolving it with an isotropic Gaussian kernel of 3 mm FWHM. A 3-mm smoothing kernel was chosen to retain finer local information within the WM tissue because DARTEL provides more accurate registration than conventional normalisation methods, and a higher smoothing value is thought to be unnecessary to compensate for suboptimal normalisation. The resulting maps representing FA were used in the group regression analysis. Through application of the mask, images that were not likely to be either WM or border areas between the WM and other tissues were removed; the FA images were not affected by signals from tissues other than WM even after smoothing. This result is important because in these areas, WM density and FA are highly correlated^12^, and the FA map should reflect the extent of WM. Furthermore, individual differences in WMC compared with other tissues can be ignored after the application of this mask because all voxels show very high WM probabilities within these masks. For validation of our preprocessing methods, see the supplemental data of Takeuchi et al.^13^.

**Statistical analysis of rWMD**

We included only voxels that showed rWMD values >0.05 in all subjects in the rWMD analyses. The primary reason for using this threshold was to reduce the periphery of the GM/WM area and effectively constrain the analysis to those areas likely to contain GM/WM. Masking the analysis to brain areas is performed by default during functional MRI (fMRI) analyses using SPM8. The GM/WM value threshold of 0.05 is a widely used value that has been reported in numerous previous VBM studies^14^.

We also investigated gender differences in rWMD and examined whether the interaction between gender and the UCLA Loneliness Scale score affected rWMD. In the whole-brain analysis, we used a voxelwise analysis of covariance (ANCOVA) in which gender was a group factor (using the full factorial option of SPM8). In this analysis, the covariates were age, RAPM score, UCLA Loneliness Scale score and TIV. All covariates, with the exception of TIV, were modelled so that each covariate’s unique relationship with rWMD was shown separately in male and female subjects (using the interactions option in SPM8), which allowed us to investigate the interactions between gender and the covariates. The TIV was modelled as having a common relationship with rWMD across genders. The interaction between gender and UCLA Loneliness Scale scores on rWMD was assessed using *t*-contrasts.

**Supplementary References**

1. Jung, R.E.*, et al.* Neuroanatomy of creativity. *Human brain mapping* **31**, 398-409 (2010).

2. Takeuchi, H.*, et al.* White matter structures associated with creativity: evidence from diffusion tensor imaging. *Neuroimage* **51**, 11-18 (2010).

3. Heinrich, L.M. & Gullone, E. The clinical significance of loneliness: a literature review. *Clin Psychol Rev* **26**, 695-718 (2006).

4. Oldfield, R.C. The assessment and analysis of handedness: the Edinburgh inventory. *Neuropsychologia* **9**, 97-113 (1971).

5. Takeuchi, H.*, et al.* Regional gray and white matter volume associated with Stroop interference: evidence from voxel-based morphometry. *Neuroimage* **59**, 2899-2907 (2012).

6. Russell, D., Peplau, L.A. & Cutrona, C.E. The revised UCLA Loneliness Scale: concurrent and discriminant validity evidence. *J Pers Soc Psychol* **39**, 472-480 (1980).

7. Sherer, M. & Adams, C.H. Construct validation of the self-efficacy scale. *Psychological Reports* **53**, 899-902 (1983).

8. Newall, N.E.*, et al.* Causal beliefs, social participation, and loneliness among older adults: A longitudinal study. *Journal of Social and Personal Relationships* **26**, 273-290 (2009).

9. Le Bihan, D.*, et al.* Diffusion tensor imaging: concepts and applications. *J Magn Reson Imaging* **13**, 534-546 (2001).

10. Takeuchi, H.*, et al.* Failing to deactivate: the association between brain activity during a working memory task and creativity. *Neuroimage* **55**, 681-687 (2011).

11. Turken, A.*, et al.* Cognitive processing speed and the structure of white matter pathways: convergent evidence from normal variation and lesion studies. *Neuroimage* **42**, 1032-1044 (2008).

12. Hugenschmidt, C.E.*, et al.* Relating imaging indices of white matter integrity and volume in healthy older adults. *Cereb Cortex* **18**, 433-442 (2008).

13. Takeuchi, H.*, et al.* White matter structures associated with empathizing and systemizing in young adults. *Neuroimage* **77**, 222-236 (2013).

14. Focke, N.K., Thompson, P.J. & Duncan, J.S. Correlation of cognitive functions with voxel-based morphometry in patients with hippocampal sclerosis. *Epilepsy & Behavior* **12**, 472-476 (2008).
